# Mouse Preclinical Cancer Immunotherapy Modeling Involving Anti-PD-1 Therapies Reveals the Need to Use Mouse Reagents to Mirror Clinical Paradigms

**DOI:** 10.3390/cancers13040729

**Published:** 2021-02-10

**Authors:** Arta M. Monjazeb, Ziming Wang, Logan V. Vick, Cordelia Dunai, Christine Minnar, Lam T. Khuat, William J. Murphy

**Affiliations:** 1 Department of Radiation Oncology, Comprehensive Cancer Center, University of California Davis School of Medicine, Sacramento, CA 95817, USA; ammonjazeb@ucdavis.edu (A.M.M.); lvvick@ucdavis.edu (L.V.V.); 2 Department of Dermatology, University of California Davis School of Medicine, Sacramento, CA 95817, USA; Ziming.Wang@Pennmedicine.upenn.edu (Z.W.); cdunai@ucdavis.edu (C.D.); ltkhuat@ucdavis.edu (L.T.K.); 3 Laboratory of Tumor Immunology and Biology, Center for Cancer Research, National Cancer Institute, Bethesda, MD 20892, USA; minnarcm@nih.gov; 4 Department of Internal Medicine, Division of Hematology and Oncology, University of California Davis School of Medicine, Sacramento, CA 95817, USA

**Keywords:** PD-1, checkpoint, immunotherapy

## Abstract

**Simple Summary:**

Immune checkpoint inhibition has revolutionized clinical cancer care. As clinical use of these inhibitors increases, the ability to study the effects of these therapies in preclinical models becomes more important. This study highlights the need for using species appropriate reagents to properly evaluate the efficacy and toxicity of cancer immunotherapy in mouse models.

**Abstract:**

Immune checkpoint inhibition (ICI) has emerged as one of the most powerful tools to reverse cancer induced immune suppression. Monoclonal antibodies (mAbs) targeting programmed cell death 1/programmed cell death ligand 1(PD-1/PD-L1) are FDA-approved and their clinical use is rapidly expanding. As opposed to the clinical paradigm, which can result in significant responses and toxicities, it has been difficult to reproduce these effects preclinically using mouse models. In large part, this is due to models, which employ rapidly growing ex vivo cultured transplantable tumor cell lines engrafted into young naïve inbred laboratory mice. However, another issue concerns the use and repeated application of xenogeneic reagents in mice (i.e., rat or hamster mAbs directed against mouse antigens at variance with clinical use of human or humanized mAbs). Building on our previous studies demonstrating that repeated administration of commonly used xenogeneic anti-PD-1 mAbs derived from both rat and hamster can induce fatal hypersensitivity in some tumor-bearing mice, we sought to compare these result with the effects of a mouse anti-mouse PD-1 mAb. Application of a murine anti-mouse PD-1 (clone: MuDX400) did not result in lethal anaphylaxis in the 4T1 tumor model. It also displayed superior antitumor effects in this and other tumor models, as it did not induce neutralizing antibody responses against the anti-PD-1 mAb, such as were observed when using xenogeneic anti-PD1 mAbs. These results demonstrate that more accurate preclinical modeling necessitates the use of mouse reagents mirroring the clinical scenario to ascertain long-term effects or toxicities, while avoiding xenogeneic responses, which do not occur clinically. Furthermore, these studies suggest a direct mechanism, whereby preclinical murine studies have often failed to recapitulate the clinical efficacy and toxicity of single agent checkpoint inhibition.

## 1. Introduction

With a deeper understanding of tumor immunology, novel therapies have been developed that seek to counteract cancer induced immune suppression and generate T cell mediated antitumor responses. Immunotherapy strategies, including vaccines, checkpoint blockades, and chimeric antigen receptor T cells have demonstrated clinical promise [1]. The recent successes of immunotherapy, particularly checkpoint blockade of the programmed cell death 1/programmed cell death ligand 1 (PD-1/PD-L1) (PD-(L)1) axis, have been tremendous breakthroughs in cancer treatment.

PD-1 is an important inhibitory receptor that regulates pathways that affect the strength and duration of immune responses to limit immune-mediated tissue damage, promote resolution of inflammation, and maintain self-tolerance [2,3]. PD-1 can be expressed on CD4^+^ T cells, CD8^+^ T cells, and B cells, as well as other cell types, such as natural killer T (NKT) cells, and activated monocytes [2,4,5,6]. Two known ligands bind to PD-1: PD-L1 and PD-L2. PD-L2 expression can be induced on dendritic cells (DCs), macrophages and cultured bone marrow–derived mast cells [7,8]. However, PD-L1 is more broadly expressed on immune cells and non-hematopoietic cells [7,9]. The ligation of PD-1 and PD-L1/2 functions as a brake to inhibit immune responses [10]. In many cancers, tumors can hijack this inhibitory checkpoint pathway to evade immune eradication [10]. PD-(L)1 blockade using monoclonal antibodies (mAbs) can lead to improved antitumor T cell cytotoxicity, proliferation, and proinflammatory cytokine production, which promote tumor destruction in many preclinical models and clinical responses in patients, although off-target effects and toxicities still remain a significant issue [3,11,12,13,14].

Given the success of checkpoint blockade, it is essential to understand the full spectrum of responses and toxicities with immunotherapies and improve the mechanistic understanding of these therapies. Preclinical mouse models using transplantable tumor cell lines have been the foundation for the study of cancer immunology and novel cancer therapies, and the pre-eminence of mouse models in cancer experimental therapeutics is unlikely to be displaced in the near future [14,15,16,17,18]. However, there are increasingly recognized limitations of mouse models, particularly when modeling cancer immunotherapy. Outside of the species disparities, the transplantable tumor models employ tumor cell lines that have often been extensively cultured ex vivo, are homogeneous and rapidly growing, and are implanted into young, healthy, immunologically naïve inbred laboratory mice. Another limitation regards the lack of mouse (i.e., species-identical) reagents used in preclinical models, which differs markedly from the clinical paradigm. The vast majority of monoclonal antibodies toward mouse determinants, including PD-1/PDL-1, used for in vivo assessment are not generated in mice, but rather in other species, such rats or hamsters, which can elicit strong immune reactions to xenogeneic antigens. We and others have observed repeated therapeutic administration of xenogeneic antibodies (other mAbs or antisera) in some mouse tumor models or even naïve mice can cause development of neutralizing antibodies and even fatal anaphylaxis [18,19]. This parallels the initial failures of using mouse antibodies clinically due to generation of neutralizing human-anti-mouse antibodies (HAMA) necessitating humanization or generation of fully human mAbs to allow for repeated application. This represents a significant hurdle for mouse cancer models to mirror treatments in human cancer patients, in which therapeutic antibodies are fully human or humanized and given for much longer periods of time. Moreover, it does not allow for assessment of long-term effects of immunotherapy and causes limitations on assessing optimal dosing and timing of repeated administration of treatments.

In this study, we report that repeated (greater than 5–6 injections over time) intraperitoneal administration of a commonly used hamster anti-mouse anti-PD-1 (αPD-1) (clone: J43) monoclonal antibody caused fatal hypersensitivity reactions in the highly inflammatory orthotopic 4T1 murine mammary carcinoma model. This fatal hypersensitivity reaction was associated with the massive infiltration of polymorphonuclear leukocytes within the lungs of anaphylactic mice. Moreover, there was an increase of specific-IgG1 antibodies in the serum. However, the application of a species-compatible mouse anti-mouse αPD-1 (clone: MuDX400) did not cause immune-mediated fatality in the 4T1 tumor model, but instead, displayed antitumor effects in both 4T1 and B16 tumor models. In contrast to the J43 clone, no induction of hamster specific-IgG1 antibodies in the serum was detected and long-term application was permissible. Our findings put the spotlight on the use of xenogeneic checkpoint blockade antibodies in mouse tumor models. Aside from differences in antitumor efficacy, they can also cause immune-related adverse effects that are not reported in humans. Our study highlights the advantage of using species-matched reagents for preclinical modeling as a key issue when investigating antibody-based cancer immunotherapy to allow for more accurate evaluation of long-term efficacy and toxicities.

## 2. Results

**Figure 1:**
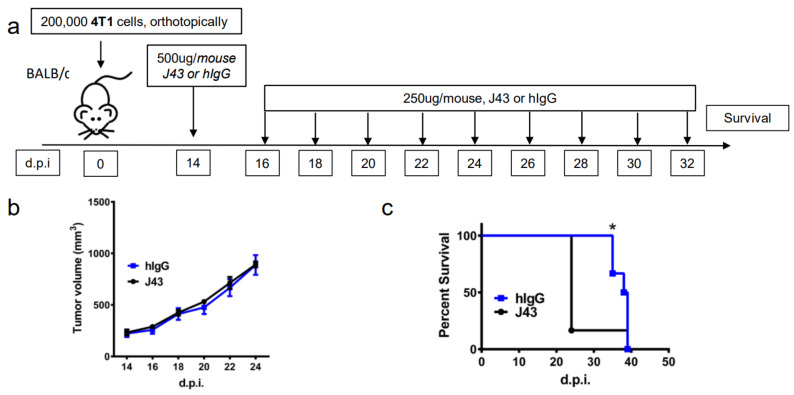

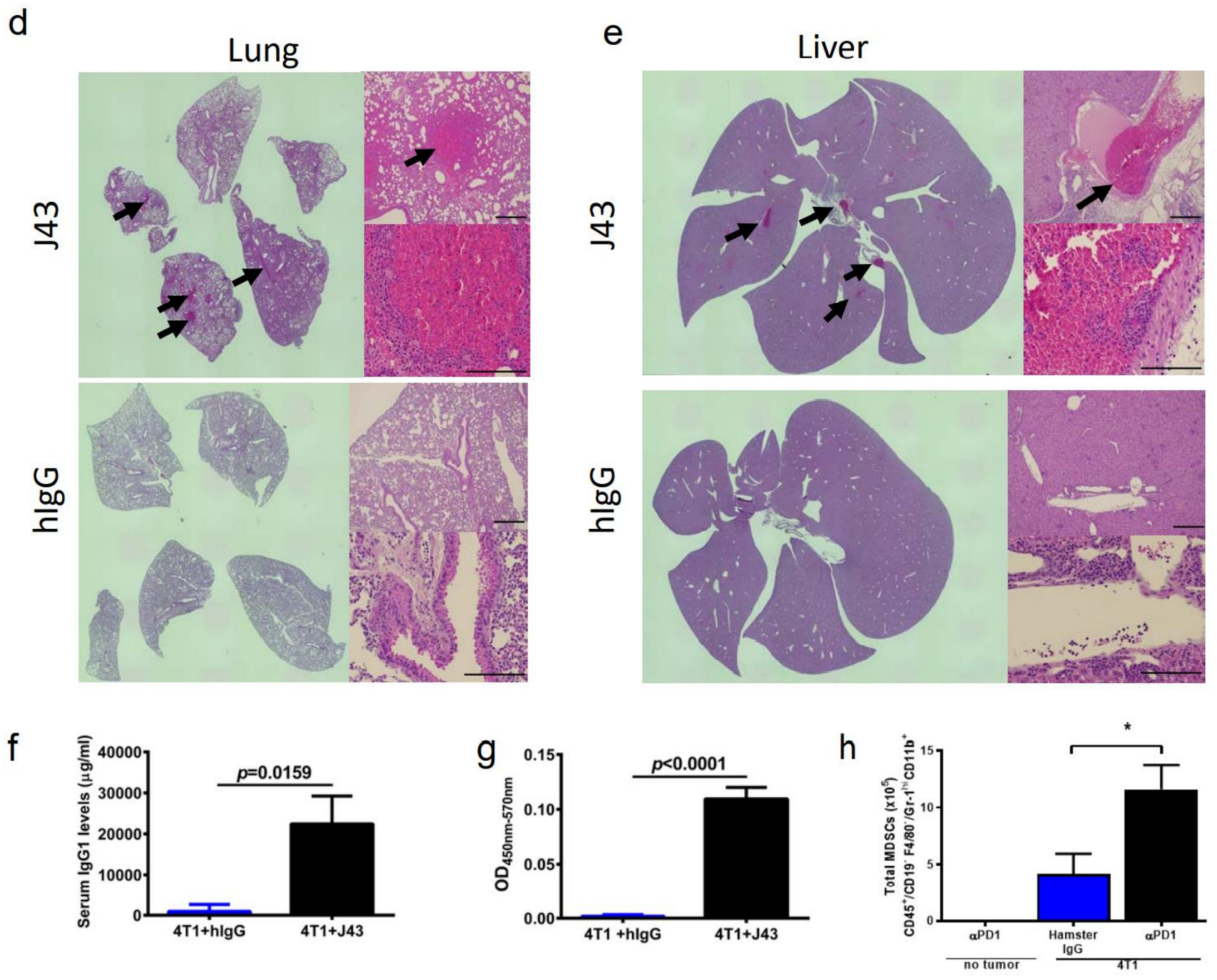
Repeated xenogeneic α programmed cell death 1 (PD-1) administration induced fatal hypersensitivity in the 4T1 breast cancer model. (**a**) Experimental schema: BALB/c mice were inoculated orthotopically with 4T1 breast carcinoma cells in the breast mammary fat pad and administered either hamster anti-mouse isotype (hIgG) or αPD-1 (clone: J43). (**b**) Tumor growth curves of 4T1 in BALB/c mice depicted as mean ± s.e.m. over days post inoculation (d.p.i.). (**c**) Kaplan–Meier survival curves of 4T1-tumor-bearing BALB/c mice treated with either hIgG or J43. Log-rank (Mantel–Cox) test used to compare groups. Representative H&E stains of (**d**) lungs and (**e**) liver of 4T1 mice treated with either hIgG or J43 demonstrating inflammation, loss of normal tissue architecture, and influx of mononuclear cells (black arrows). (**f**) Serum IgG1 levels in mice treated with either hIgG or J43. (**g**) J43 reactive IgG1 levels in the serum of 4T1 tumor-bearing mice treated with either hIgG or J43. (**h**) Absolute numbers of myeloid derived suppressor cells (MDSCs) in the lungs of 4T1 tumor bearing mice as assessed by flow cytometry. Bar graphs represent the mean value and error bars represent the standard error of the mean. * *p* < 0.05, *n* = 6 mice per group.

Repeated xenogeneic αPD-1 administration induces fatal hypersensitivity in the inflammatory 4T1 breast cancer model. The 4T1 is a commonly used model of triple-negative human breast cancer and can spontaneously metastasizes to lungs, liver, brain, and bone [20,21,22,23]. It has previously been reported by our group that 4T1 tumor-bearing mice will succumb to anaphylaxis after the repeated administration of xenogeneic hamster or rat αPD-1 monotherapy but not with xenogeneic species-specific antibody controls or xenogeneic anti- cytotoxic T-lymphocyte-associated protein 4 (CTLA4) mAb [18]. This highlights the unique aspects of PD-1/PDL-1 as an immunological adjuvant target. Interestingly, this anaphylaxis was not observed using non-tumor bearing mice, or with other tumor models or strains, and is likely also dependent on the number of injections as well as amounts delivered [18]. These results suggest that the anaphylaxis is due to both the inflammatory nature of 4T1 in combination with the inhibition of PD-1. Building on these studies, we implanted 4T1 murine mammary carcinoma orthotopically into the breast mammary pad of female BALB/c mice to further examine the effects of xenogeneic αPD-1 in this model. The tumor-bearing mice were then treated with repeated injections of either αPD-1 (hamster anti-mouse, clone: J43) or isotype control (hamster IgG, hIgG) as shown in the experimental schema (Figure 1a). The J43 mAb did not result in significant antitumor effects due to the limited capability to administer the mAb, which caused fatal and rapid anaphylaxis following the fifth–sixth injection. The tumor volumes were comparable between the hIgG and J43 groups (Figure 1b). After repeated anti-PD-1 administration, the mice became cyanotic with labored breathing 30–60 min following the fifth–sixth injection resulting in rapid death (Figure 1c). It is important to note that these effects only occurred after prolonged administration and are contingent on the frequency and amount of mAb administered, strain of mouse (BALB/c) and existence of the inflammatory tumor which is noted for high myeloid-derived suppressor cells (MDSC) content. These symptoms have been described in hypersensitivity reactions and have been observed in other models such as repeated administration of xenogeneic antisera (i.e., rabbit anti-mouse asialo GM1, ASGM1, antisera used to deplete NK cells) in mice [18,24,25,26]. Immune hypersensitivity is characterized by increased vascular permeability, respiratory arrest due to smooth muscle contraction, multi-organ failure, or cardiac arrest due to decreased cardiac output [27,28]. We assessed pathology in the lungs and livers. Neutrophils accumulated in lungs of 4T1 mice treated with J43 but not in the lungs of hamster IgG-treated mice. We observed marked leukostasis within interstitial alveoli and bronchioli (Figure 1d). Livers of J43-treated 4T1-bearing mice also exhibited accumulation of infiltrated polymorphonuclear (PMN) leukocytes (Figure 1e). The 4T1 tumor model is well-characterized for the massive expansion of MDSC, both systemically and within the tumor. We previously demonstrated that removal of the MDSCs could protect tumor-bearing mice from the anaphylactic death [18]. The IgG1 levels in serum were collected and measured by ELISA after the injection. The serum mIgG1 levels were significantly higher in the 4T1 tumor-bearing mice treated with J43 than those in the hamster IgG group (Figure 1f), suggesting that the αPD-1 activity of J43 increased the mouse anti-hamster response greater than injections of control hamster IgG. We next determined the levels of mIgG1 specific for J43. ELISA data showed that there was an increase in anti-J43 mIgG1 antibodies in 4T1 tumor-bearing mice receiving αPD-1 therapy (Figure 1g), which indicated xenogeneic αPD-1 treatment can cause hypersensitivity by increasing the sera levels of αPD-1 specific IgG1 antibodies. Hamster IgG did not significantly induce neutralizing antibodies in the 4T1 mouse model (Figure 1g). These results indicate induction of anti-hamster antibodies mediates hypersensitivity reactions in 4T1 tumor-bearing BALB/c mice receiving prolonged αPD-1 monotherapy. These results cannot be attributed to use of xenogeneic mAbs alone, as the hamster IgG controls did not have similar responses. Nor are such responses seen in non-tumor bearing mice treated with the αPD-1 [18]. Thus, the presence of PD-1 blockade in combination with the repeated xenogeneic exposure and the inflammatory tumor conditions resulted in the lethal toxicity. As expected given the inflammatory nature of 4T1, MDSC levels were higher in the lungs of tumor bearing mice than non-tumor bearing mice (Figure 1h). These levels were significantly increased in 4T1-bearing mice treated with xenogeneic αPD-1 compared to hIgG (Figure 1h). This suggests that increased MDSCs induced by 4T1 may contribute to the anaphylactic responses and we have previously shown that depletion of GR-1+ cells can reduce the toxicity seen in this model [18]. This anaphylaxis obviated any attempt to ascertain potential antitumor effects but also indicated that neutralizing xenogeneic Abs are being induced in the mice likely limiting efficacy as well.

**Figure 2:**
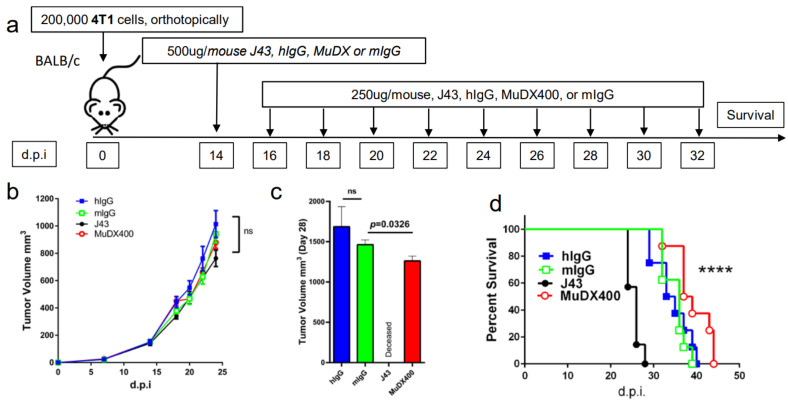

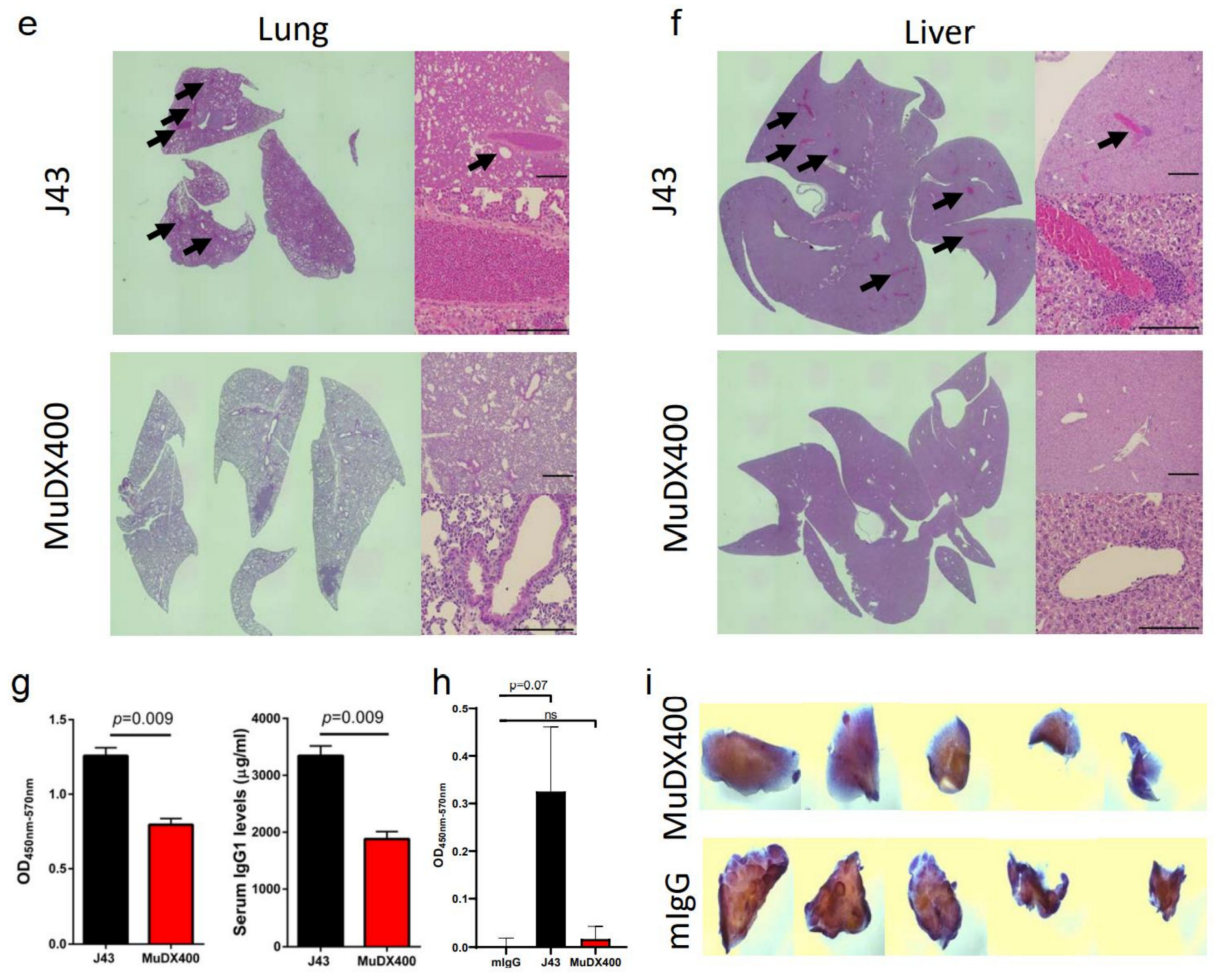
Murine αPD-1 avoided the fatal hypersensitivity associated with xenogeneic reagent in 4T1 breast cancer model. (**a**) Schema showing BALB/c mice were inoculated with 4T1 breast carcinoma cells orthotopically in the breast mammal pad and followed by monotherapy with either mouse αPD-1 (MuDX400) or J43. (**b**) Tumor growth curves of 4T1 in BALB/c mice. Tumor volumes depicted as mean ± s.e.m. (**c**) Tumor volume of 4T1 in BALB/c mice at day 28. Tumor volumes depicted as mean ± s.e.m. (**d**) Kaplan–Meier survival curves of 4T1-tumor-bearing BALB/c mice treated with either MuDX400 or J43. Log-rank (Mantel–Cox) test used to compare groups. Representative H&E stains of (**e**) lungs and (**f**) liver of 4T1 mice treated with either J43 or MuDX400 demonstrating inflammation, loss of normal tissue architecture, and influx of mononuclear cells (black arrows). (**g**) Total serum IgG1 levels evaluated via ELISA of 4T1 tumor bearing mice treated with J43 or MuDX400. (**h**) J43 reactive IgG levels in the serum of 4T1 tumor-bearing mice treated. (**i**) Representative staining of whole-mount lung to examine metastases in 4T1 mice treated with either MuDX400 or mIgG1. Bar graphs represent the mean value and error bars represent the standard error of the mean. **** *p* < 0.0001, *n* = 7 to 12 mice per group.

Use of murine anti-mouse PD-1 (MUDX400) does not result in fatal hypersensitivity associated with xenogeneic αPD-1 mAb in 4T1 breast cancer model. Due to the anaphylactic effects of the xenogeneic αPD-1 monoclonal antibodies, which are commercially available and widely used in in-vivo models, it was not possible to test the antitumor effects of long-term administration of αPD-1 in the 4T1 breast cancer model. Therefore, we used a completely murine αPD-1 (MuDX400) in the 4T1 model and monitored for anaphylaxis and efficacy. We compared tumor-bearing mice treated with J43 versus MuDX400 (Figure 2a). Checkpoint inhibition with αPD-1 (J43 and MuDX400) did not show differences in primary 4T1 tumor growth compared to controls up to the sixth injection at day 24, which is not surprising as in most mouse tumor studies, anti-PD-1 as a monotherapy yields modest to negligible effects due to the rapid growth of mouse tumor lines in vivo (Figure 2b). The 4T1 tumor-bearing mice treated with J43 demonstrated toxicity starting after the sixth injection and 100% of mice had lethal anaphylaxis by the eighth injection (Figure 2c,d). In marked contrast, mice tolerated long-term administration of MuDX400 and began to demonstrate a modest but statistically significant improvement in tumor growth and survival compared to controls (Figure 2c,d). MuDX400-treated 4T1 tumor-bearing mice were administered 10 injections with no signs of toxicity. By 34 d.p.i. they were sacrificed due to progression of tumors (Figure 2d) and displayed no symptoms of hypersensitivity reaction., Unlike the J43-treated mice which showed lung and liver pathology (Figure 1d,e and Figure 2e,f) there were no signs of lung and liver pathology in the MuDX400-treated 4T1 tumor-bearing mice (Figure 2e,f). We were able to confirm that total mIgG1 levels were increased in mice treated with J43 when compared to the murinized MuDx400 (Figure 2g). Furthermore, to clarify specificity, repeated MUDX400 administration did not result in the induction of an antibody response to hamster J43 protein determinants as compared to J43 treated mice (Figure 2h). We then assessed for effects on 4T1 metastases after the sixth treatment by staining whole-mount lungs (Figure 2i). The lungs of isotype control treated 4T1 tumor-bearing mice presented with numerous metastases, which resulted in significant lung pathology. However, the MuDX400-treated 4T1-bearing mice displayed less lung metastases compared to the isotype controls and retained a grossly normal anatomical structure (Figure 2i). Overall, the use of the mouse αPD-1 monoclonal antibody did not cause anaphylaxis and resulted in significant antitumor effects, particularly in preventing lung metastases. MuDX400 treatment resulted in improved survival in the 4T1 mouse model, a model that so far had not been ameliorated by checkpoint blockade. These results demonstrate that the use of a mouse reagent obviates toxicities observed when conventional anti-PD-1 mAbs are used, and suggests that anaphylaxis is not reflective of the clinical paradigm where humanized mAbs are routinely used.

**Figure 3:**
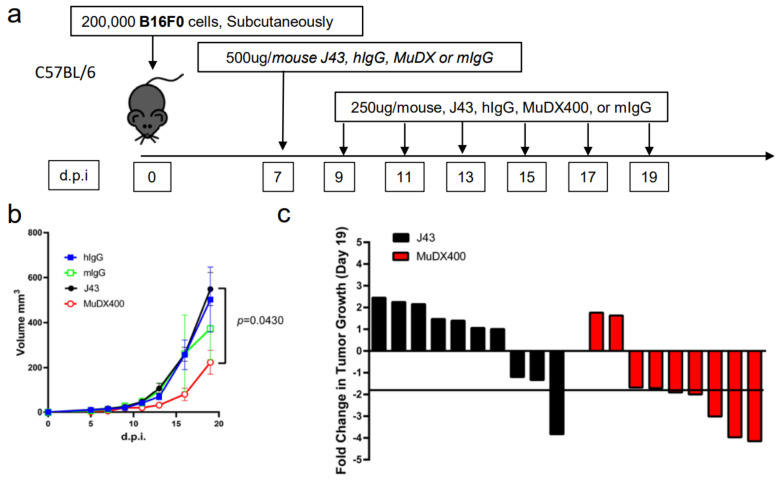

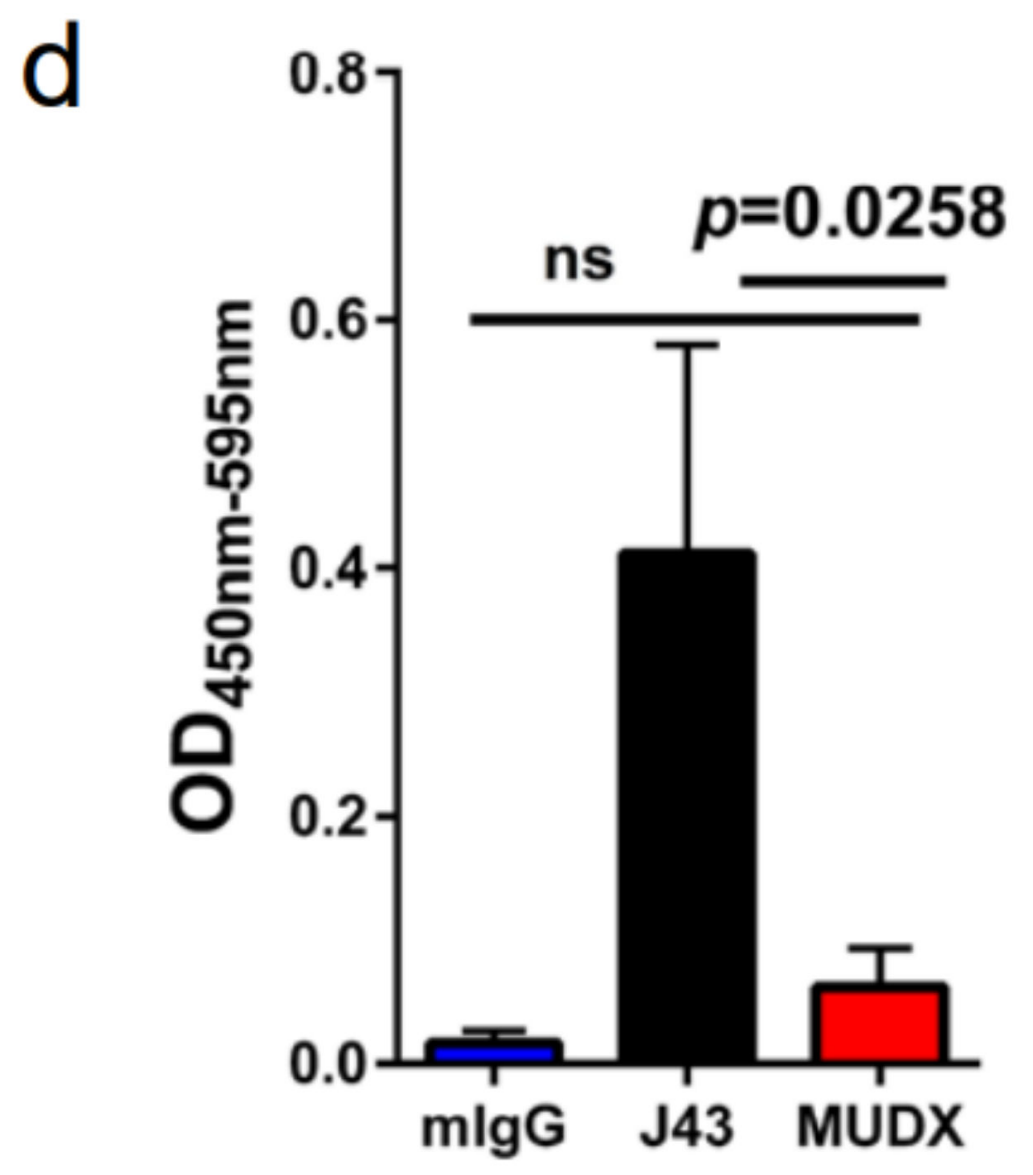
Repeated murine αPD-1 treatment displayed antitumor efficacy in the B16 melanoma model. (**a**) Experimental schema: C57BL/6 mice were inoculated with B16-F0 melanoma cells subcutaneously and administered with either J43 or MuDX400. (**b**) Tumor growth curves of B16-F0 in C57BL/6 mice. Tumor volumes depicted as mean ± s.e.m. (**c**) Waterfall plot of pooled data demonstrating fold change in tumor growth compared to IgG treated controls. (**d**) ELISA assessment of J43 reactive IgG antibodies in the serum of B16-F0 tumor bearing mice with or without treatment. Bar graphs represent the mean value and error bars represent the standard error of the mean. *n* = 9 to 11 mice per group.

Repeated murine αPD-1 treatment displayed antitumor efficacy in the B16-F0 melanoma model. We have previously reported that in tumor models, which are less inflammatory than 4T1, such as Renca (renal carcinoma cell line) in BALB/c mice, or B16 melanoma or 3LL tumors in other mouse strains such as C57BL/6, we did not observe similar anaphylactic responses to repeated xenogeneic αPD-1 treatment [18]. However, the absence of anaphylaxis observed when these antibodies are repeatedly applied in other mouse tumor models does not mean that potential neutralizing antibodies are not induced. We therefore assessed the efficacy of and presence of neutralizing antibodies to murine vs. xenogeneic anti-PD-1 mAb. αPD-1 was first approved for treating metastatic melanoma in humans. However, αPD-1 monotherapy in mice has failed to recapitulate the impressive antitumor responses seen in the clinic, particularly in the B16 melanoma model. We examined the effects of murine vs. xenogeneic αPD-1 in B16 tumor-bearing mice using either J43 or MuDX400 as shown in the schema (Figure 3a). B16 tumor-growth progressed rapidly in J43 and control groups with no significant differences between these groups, which is consistent with previous reports by us and others. However, the tumor sizes of B16 tumor-bearing mice with MuDX400 treatment were significantly reduced compared to mice treated with J43 αPD-1 (Figure 3b,c). In examining the individual tumor growth pooled from two experiments we found that only 1/10 J43 treated mice had a greater than 1.5-fold reduction in tumor growth compared to controls, whereas 7/9 MuDX400 treated mice displayed this level of tumor growth reduction (Figure 3c). The reduced efficacy of xenogeneic αPD-1 in comparison to murine αPD-1 may indicate that the immune system produces neutralizing antibodies against the xenogeneic agent, which detrimentally affects the pharmacokinetics. Indeed, the ELISA results demonstrated increased levels of IgG directed against hamster antibodies in the sera of J43-treated mice but not in MuDX400-treated mice or control mIgG treated mice (Figure 3d). These results demonstrate that use of species-compatible reagents can be critical for long-term application and assessment in mouse immunotherapy models.

## 3. Discussion

In the current study, we show that repeated dosing with xenogeneic PD-1 mAbs induces rapid fatal hypersensitivity in 4T1 tumor-bearing mice but that this does not occur in a less inflammatory tumor models or with xenogeneic isotype controls. This suggests that this fatal xenogeneic hypersensitivity reaction is likely a synergistic inflammatory effect of PD-1 blockade, the xenogeneic antibody, and the 4T1 model. This is evidenced in part by the increase in lung MDSCs observed in 4T1 tumor bearing mice treated with xenogeneic αPD-1, which exceeds the levels seen in non-tumor bearing mice treated with xenogeneic αPD-1 or in 4T1 tumor bearing mice treated with xenogeneic isotype control antibodies. It is important to note that this type of fatal xenogeneic reaction can occur in the absence of these particular conditions since repeated administration of xenogeneic antisera (i.e., rabbit antisera to asialo GM1 in mice) has been well-documented to also induce anaphylaxis after repeated injections, even in naive mice. The hypersensitivity reactions to xenogeneic αPD-1 was further evidenced by the increased levels of IgG1 antibodies in serum and severe pathology in both lungs and livers, which occurred after 5–6 injections [18,29]. Importantly, while fatal anaphylaxis was only observed using the 4T1 tumor model, the induction of neutralizing antibodies against the xenogeneic antibodies can potentially impact efficacy in any tumor model. We observed superior responses using a mouse αPD-1 antibody when using a prolonged treatment schedule with multiple administrations. This was highlighted by the data demonstrating that the murine anti-mouse PD-1 (MuDX400) exhibited antitumor effects in both the 4T1 and B16 tumor models, which were not observed with hamster αPD-1 (J43). The two different clones of αPD-1 are behaving as two different drugs in terms of the toxicity and efficacy profile, despite targeting the same molecule. While we have previously shown similar results in regards to anaphylaxis with other xenogeneic αPD-1 clones [18], confirmation of our current findings using additional clones and in additional tumor models is warranted. It is possible that differences can be partly attributed to the recognition of different epitopes on PD-1 by these antibodies; however, other commercially available xenogeneic αPD-1 antibodies have also demonstrated limited efficacy in these models. A more plausible mechanism for these differences is the generation of neutralizing antibodies against the xenogeneic αPD-1. These issues could also be critical when investigating mechanisms of acquired resistance to checkpoint inhibitors as occurs in the clinical setting. Our sera IgG1 ELISA data demonstrated increased levels of circulating IgG1 and increased levels of IgG1 directed against hamster IgG after use of xenogeneic αPD-1. Thus, neutralizing antibodies generated by the immune system upon the introduction of xenogeneic αPD-1 likely influence the pharmacokinetics of αPD-1 and alter efficacy. However, MuDX400 is a mouse anti-mouse IgG1 monoclonal antibody, which created a barrier to quantifying the neutralizing antibodies in the serum of mice treated with MuDX400 by ELISA. In order to further confirm our hypothesis, the concentrations of the αPD-1 antibody therapies in the blood should be measured by mass spectrometry.

MuDX400 administration demonstrated antitumor effects on primary tumor progression and lung metastases. However, preclinical tumor cell lines, 4T1 and B16, may grow too fast to fully observe the promising antitumor effects which have been seen in clinical patients. This highlights the difficulty of modeling clinical kinetics in mice. The use of genetically engineered models or indolent tumor models may therefore be more relevant and advantageous. These enable slower tumor progression with potential observation of therapeutic effects. Indolent models would also facilitate the long-term assessment of these drugs for immune related adverse effects possible.

These studies highlight the limitations of studying checkpoint blockade in mouse tumor models. In clinical studies employing mouse mAbs, the generation of human-anti-mouse antibodies (HAMA) to the xenogeneic proteins markedly impaired efficacy, necessitating conversion to human/humanized mAbs to enable repeated application. The emergence and use of mouse αPD-1 monoclonal antibodies such as MuDX400 will help preclinical studies more closely model the clinical scenario and improve our understanding of checkpoint inhibition. Additionally, as PD-1 targeted therapies are being increasingly applied for long periods of time (up to years), as well as being used in combination with other therapies, it will be critical for preclinical studies to better mirror the clinical paradigm when assessing efficacy and toxicities. It is likely that other checkpoint pathways/molecules being similarly targeted by xenogeneic agents in murine models also induce neutralizing responses, leading to hypersensitivity or limiting evaluation of toxicity and efficacy.

## 4. Materials and Methods

### 4.1. Mice

Female BALB/cAnNCrl of 6 weeks old were purchased from Charles River Laboratories, Inc. Female BALB/cAnNTac and C57BL/6NTac, as well as male C57BL/6NTac of 4–6 weeks old were purchased from Taconic Farms. Mice were housed in AALAC-accredited animal facilities at University of California Davis (UC Davis, Sacramento, CA, USA) under specific-pathogen-free conditions. Protocols were approved by UC Davis IACUC and studies complied with ethical regulations and humane endpoints (approval numbers 20680 and 20707).

### 4.2. Tumor Cell Line and Treatment

The murine breast cancer cell line 4T1 (CRL-2539) and melanoma cell line B16-F0 (CRL-6322) were obtained from the American Type Culture Collection. BALB/c mice were injected subcutaneously in the right mammary pad with 2 × 10^5^ 4T1 tumor cells in 100 μL PBS. C57BL/6 mice were injected subcutaneously in the right flank with 2 × 10^5^ B16-F0 cells in 100 μL PBS. Tumor growth was monitored daily and measured every 2–3 days. Tumor volume was determined as length (mm) × width^2^ (mm) × 0.5. Tumor bearing mice were treated intraperitoneally with 500 μg αPD-1 monoclonal antibody (clone: J43, BioXCell) in 200 μL PBS, or αPD-1 monoclonal antibody (clone: 03AHF, MuDX400 from Merck, Kenilworth, NJ, USA) in acetate and sucrose buffer (NaAc) provided by Merck on day 14 post tumor inoculation (d.p.i.), followed by 250 μg αPD-1 monoclonal antibody every other day continuously. Control mice received Syrian Hamster Gamma Globulin (Jackson ImmunoResearch, West Grove, PA, USA) in 200 μL PBS, or mouse IgG1 (clone: 61AVY, Merck) in 200 μL 20Mm NaAc. Mice were euthanized when they were moribund with hypersensitivity (immobile, prostrate position, cyanotic with labored breathing).

### 4.3. Mouse Lung Whole-Mount Preparation

Lungs from 4T1-bearing BALB/c mice were first flushed by PBS, then separated into five lobes, collected, and fixed in 10% neutral buffered formalin. Lung tissues were transferred to 70% alcohol for 2 h then to 100% alcohol for another 2 h. Lungs were then dehydrated using three changes of xylene (30 min, 1 h, 1 h), followed by processing through a graded series of alcohol. After rinsing in running tap water for 30 min, the tissues were stained with hematoxylin for 2 min. Lungs were de-stained in a 1% HCl solution for 15 min, then placed under running tap water for 30 min, 70% alcohol for 1 h, 100% alcohol for 1 h, and, finally, xylene for 1 h. Whole mounts were then submerged in methyl salicylate for storage.

### 4.4. H&E Staining

Lungs and livers from 4T1-bearing BALB/c mice and B16-F0-bearing C57BL/6 mice were fixed in 10% paraformaldehyde and embedded in paraffin. Multiple 4 μm sections were cut for H&E staining. Slides were prepared and stained at in the UC Davis Pathology Core. Images were captured by a BioRevo BZ-9000 Fluorescence Microscope (Keyence, Osaka, Japan); 9–18 images of each specimen were taken in Brightfield, at 2× magnification and 1/80 s exposure. Then, images were stitched together to generate whole-specimen images via accompanying software Bio-Analyzer (Keyence, Osaka, Japan).

### 4.5. ELISA

The concentrations of serum IgG1 levels and anti-J43 IgG1 levels were measured by using mouse IgG1 ELISA Ready-SET-GO!^®^ kit from eBioscience. Moreover, 96-well high affinity protein binding plates (Corning Costar 9018) were coated with 100 µL pre-titrated, purified anti-mouse IgG1 monoclonal antibody or 100 µL of J43 (10 µg/mL) and incubated at 4 °C overnight. Wells were aspirated and washed twice with 400 µL Wash Buffer. Plates were washed for 1 min during each wash step. Wells were blocked with 250 µL Blocking Buffer and incubated at 4 °C overnight. Wells were aspirated and washed again. A total of 100 µL standards and 50 µL prediluted serum samples were added to plates, 50 µL Assay Buffer was added to sample wells; 50 µL diluted Detection Antibody was added to each well. Plates were covered and incubated at room temperature for 3 h. Wells were aspirated and washed 4 times. A total of 100 µL TMB Substrate Solution was added into each well and incubated for 15 min. Absorbance was read at 450 nm and 570 nm on the VersaMax^TM^ Tunable microplate reader (VWR, Radnor, PA, USA) using the SoftMax Pro Data Acquisition and Analysis Software (Molecular Devices Corp., Sunnyvale, CA, USA). The concentrations of serum IgG1 levels were obtained by subtracting the values of 570 nm from those of 450 nm. Absolute changes in OD were calculated by subtracting out levels in control PBS treated mice.

### 4.6. Statistics

Survival data were plotted by the Kaplan–Meier method and compared by log-rank test. Data were expressed as mean ± s.e.m. One-way or 2-way ANOVA or Student’s *t*-tests were used to compare groups. Graph-Pad Prism 6 Software was used to perform the statistical analyses (GraphPad Software, Inc., La Jolla, CA, USA). Unless otherwise identified significance indicates: **** *p* < 0.0001, *** *p* < 0.001, ** *p* < 0.01, and * *p* < 0.05.

## 5. Conclusions

Xenogeneic αPD-1 can induce fatal hypersensitivity in certain inflammatory tumor models, such as 4T1. This appears to be dependent on a highly inflammatory tumor model, the xenogeneic reagent, and the αPD-1 activity of the reagent. This is not observed with other tumor models, with the use of xenogeneic control IgG, or with the use of mouse αPD-1. Thus, the use of mouse αPD-1 allows for evaluation of long-term administration of therapy in the 4T1 model and demonstrates modest antitumor effects of the therapy in this model. In other models where anaphylaxis is not observed, the use of xenogeneic reagents still limits studies. Induction of neutralizing antibodies against the xenogeneic αPD-1 antibody limits efficacy. In the B16 model, hamster αPD-1 induces specific antibodies over time and has limited efficacy, whereas murine αPD-1 is amenable to prolonged administration and demonstrates robust antitumor effects. The use of species-specific reagents is critical to evaluating the efficacy and toxicity of antibody-based cancer immunotherapy in mouse models.

## Data Availability

The data presented in this study are available in this article.
